# Online Bias Estimation for Single-Platform Airborne Radar Using Bias-Subspace Information-Guided MAP-EKF

**DOI:** 10.3390/s26155005

**Published:** 2026-08-06

**Authors:** Junwu Luo, Xujun Guan, Chuang Song, Hai Zhang

**Affiliations:** 1 School of Automation Science and Electrical Engineering, Beihang University, Beijing 100191, China; luojunwu@buaa.edu.cn (J.L.); zhanghai@buaa.edu.cn (H.Z.); 2 National Key Laboratory of Complex System Control and Intelligent Agent Cooperation, Beijing 100074, China; 13381055619@163.com

**Keywords:** single-platform radar, systematic bias estimation, weak observability, MAP-EKF

## Abstract

Systematic measurement biases are a persistent source of degradation in airborne radar target tracking. Online bias estimation is especially difficult for single-platform operations because only one measurement stream is available, and the target state and radar biases are coupled in the same nonlinear observation model. Under weakly observable geometries, directly augmenting bias states into a recursive filter may lead to slow convergence, while fixed or overly frequent batch optimization may inject updates from weakly informative windows. To address this problem, this paper proposes a Bias Subspace Information-Guided MAP-EKF (maximum a posteriori–extended Kalman filter) (BI-MAP-EKF) for online bias estimation in single-platform airborne radar. A nine-dimensional augmented state jointly describes target motion and range, azimuth, and elevation biases. A posterior Cramér–Rao lower bound (PCRLB) is constructed for this state, where the EKF prior, windowed radar measurements, and process noise propagation are jointly considered. The bias subspace is then extracted by Schur complement, and the resulting azimuth bias PCRLB is used to decide whether the MAP update is reliable enough for EKF injection. The scheduler also includes a cooldown interval and a maximum-window safeguard, which respectively limit excessive updates under informative maneuvers and prevent indefinite waiting under weak geometries. Fifty-run Monte Carlo simulations under different maneuvering conditions show that the proposed scheduler is particularly effective in weakly informative geometries, where it improves azimuth bias and horizontal position estimation while reducing the number of accepted MAP refinements compared with the tested fixed-period MAP and moving-horizon estimation (MHE) baselines. In stronger maneuvering scenarios, it provides a competitive accuracy–cost trade-off rather than uniformly outperforming aggressive MHE in every channel.

## 1. Introduction

Airborne radar is a remote sensing payload for unmanned aerial vehicle (UAV) platforms, providing range, angular, and Doppler measurements when optical sensing is unreliable [1,2]. In such radar sensing systems, the measurements are affected not only by random noise and target maneuver uncertainty, but also by systematic errors caused by antenna installation, platform attitude errors, timing offsets, and signal processing delays. These biases are sensor-level errors, but once they enter a recursive tracking estimator, they are propagated together with the target state and can lead to persistent localization errors [3,4]. The problem studied in this paper is therefore an online radar measurement calibration and sensor data fusion problem rather than a separate offline calibration task.

Most existing bias estimation and sensor registration methods are developed for multi-sensor or cooperative settings. Representative works have addressed coordinate alignment, asynchronous bias estimation, least-squares registration, and performance bounds for cooperative sensing systems [5,6,7]. In these settings, redundant observations, common targets, and distributed geometric constraints can be used to separate sensor biases from target states [8,9]. Related calibration problems have also been studied in multi-modal sensing systems, including radar–camera and camera–LiDAR–IMU calibration [10,11,12,13]. Motion geometry-based decoupling also appears in moving-camera perception, where Ma et al. [14] extracted principal motion from optical flow to separate camera ego motion from foreground motion.

This redundancy is not available in the single-platform airborne radar case considered here. The target is observed only by one onboard radar, and no auxiliary radar, cooperative transponder, or external calibration sources are assumed. As a result, the target state and the radar biases must be inferred from the same nonlinear measurement stream.

The main difficulty appears in the angular channels. A small azimuth bias can be absorbed by a cross-track displacement of the target estimate, so different state–bias combinations may explain nearly the same measurements over a limited interval. Similar geometry-dependent identifiability issues have been reported in bearings-only tracking and target motion analysis, where observer maneuvering is required to improve state distinguishability [15,16,17,18]. Although the extended Kalman filter (EKF), unscented Kalman filter (UKF), and cubature Kalman filter (CKF), as well as particle filters, are widely used in nonlinear target tracking [19,20,21,22], augmenting the bias terms into the filter state does not by itself remove this ambiguity. In weakly observable geometries, the filter may remain numerically stable while converging slowly or settling near a coupled state–bias solution.

This difficulty is fundamentally related to time-varying observability. Classical nonlinear observability theory shows that state distinguishability depends on the system dynamics and measurement functions [23]. From a statistical viewpoint, the Fisher Information Matrix (FIM) and Cramér–Rao-type bounds quantify how sensing geometry limits achievable estimation accuracy [24,25,26]. Sliding-window methods, including moving-horizon estimation (MHE) and factor graph smoothing, exploit temporal constraints beyond a single recursive update [27,28,29,30,31,32,33]. However, more data do not necessarily imply more usable information. An informative window can help separate the bias from the target state, whereas poor geometry may only reinforce the existing ambiguity. Thus, the issue is whether maximum a posteriori (MAP) refinement is reliable enough for injection into the recursive EKF.

Motivated by this observation, this paper proposes a Bias Subspace Information-Guided MAP-EKF (BI-MAP-EKF) framework for online bias calibration in a single-platform airborne radar sensing system. The recursive augmented-state EKF remains responsible for real-time estimation and covariance propagation, while MAP refinement is used only as an intermittent sensor data fusion update. The trigger is not determined by elapsed time alone. Instead, a posterior Cramér–Rao lower bound (PCRLB) is constructed for the candidate window from the EKF prior, windowed radar measurements, and process noise propagation. The bias subspace bound is extracted by Schur complement, and MAP is injected only when the azimuth bias bound indicates that the candidate window is suitable for a reliable bias update. A cooldown interval and a maximum-window safeguard regularize the injection frequency.

The main contributions of this paper are summarized as follows:

1. The online radar measurement bias calibration problem is formulated for a single-platform airborne radar, where angular biases and target position are strongly coupled under weak geometric excitation.
2. A complete posterior window PCRLB is constructed for MAP window evaluation. Compared with the deterministic backward propagation FIM, the revised formulation incorporates process noise propagation and the EKF prior, so that the bias subspace bound is consistent with the actual MAP-EKF injection problem.
3. A PCRLB-guided, resource-aware MAP-EKF scheduler is proposed. The scheduler combines an azimuth bias PCRLB threshold, a cooldown interval, and a maximum-window safeguard, allowing MAP refinement to be scheduled according to posterior bias subspace information rather than a fixed period alone.
4. Monte Carlo simulations with MHE and periodic MAP baselines show that the proposed scheduler is especially effective in weakly informative geometries, improving azimuth bias and horizontal position estimation with fewer MAP refinements. In stronger maneuvering geometries, it maintains competitive accuracy with reduced MAP calls, rather than claiming uniform dominance in every channel.

The remainder of this paper is organized as follows. Section 2 formulates the augmented state-space model and the nonlinear measurement equations. Section 3 presents the proposed BI-MAP-EKF framework, including the coupling analysis, the recursive PCRLB for maneuver-level diagnosis, and the complete posterior window PCRLB for online MAP triggering. Section 4 evaluates observability characteristics under different maneuvering modes and reports the proposed method’s performance with parameter sensitivity analysis. Section 5 provides comparative simulation results with MHE and periodic MAP baselines. Section 6 discusses the limitations of the current approach. Section 7 concludes the paper.

## 2. Problem Formulation

We consider an airborne radar mounted on a UAV platform tracking a ground object. The radar operates in a spherical coordinate system and provides four types of measurements at each sampling instant: slant range $r_{m}$, azimuth angle $\theta_{m}$, elevation angle $\varphi_{m}$, and radial velocity $v_{rm}$ (derived from Doppler shift). The following assumptions are adopted throughout this work:

- The UAV platform’s own position $p_{u,k}={\left[x_{u},y_{u},z_{u}\right]}^{T}$ and velocity $v_{u,k}={\left[v_{xu},v_{yu},v_{zu}\right]}^{T}$ are known from onboard inertial navigation system (INS)/global navigation satellite system (GNSS) data at time *k*.
- The target is a low-dynamic ground object; its motion is adequately described by the Constant Velocity (CV) model.
- The radar measurements are corrupted by both zero-mean Gaussian random noise and unknown systematic biases Δr (range), Δθ (azimuth), and Δφ (elevation). These biases are assumed constant or slowly varying over the tracking interval.
- Only a single airborne platform is available; no cooperative transponder, auxiliary radar, or multi-sensor redundancy can be exploited.

Under these assumptions, the unknown systematic biases are augmented into the target kinematic state to form a joint estimation vector. The target state in Cartesian coordinates is $x_{t}={\left[\begin{array}{cccccc} x & y & z & v_{x} & v_{y} & v_{z} \end{array}\right]}^{T}$ and the bias vector is $b={\left[\begin{array}{ccc} \Delta r & \Delta \theta & \Delta \varphi \end{array}\right]}^{T}$. The 9-dimensional augmented state is defined as

(1) $$X={\left[\begin{array}{cc} x_{t}^{T} & b^{T} \end{array}\right]}^{T}={\left[\begin{array}{ccccccccc} x & y & z & v_{x} & v_{y} & v_{z} & \Delta r & \Delta \theta & \Delta \varphi \end{array}\right]}^{T}$$

The discrete-time state dynamics follow

(2) $$X_{k}=AX_{k-1}+w_{k-1}$$

where $w_{k-1}∼N\left(0,Q\right)$ is the process noise. The state transition matrix is block-diagonal in the kinematic and bias subspaces:

(3) $$A=\left[\begin{array}{ccc} I_{3\times 3} & \Delta t\cdot I_{3\times 3} & 0_{3\times 3}\\ 0_{3\times 3} & I_{3\times 3} & 0_{3\times 3}\\ 0_{3\times 3} & 0_{3\times 3} & I_{3\times 3} \end{array}\right]$$

where Δt is the sampling interval. The biases are modeled as a random walk with small process noise intensity to accommodate slow environmental drifts. Since the kinematics are expressed in Cartesian coordinates and the bias subspace evolves as a random constant, the prediction step is strictly linear and does not introduce linearization errors [19].

Let $\Delta p=p_{t,k}-p_{u,k}={\left[\Delta x,\Delta y,\Delta z\right]}^{T}$ and $\Delta v=v_{t,k}-v_{u,k}={\left[\Delta v_{x},\Delta v_{y},\Delta v_{z}\right]}^{T}$ denote the relative position and velocity vectors. Define the slant range $R=\sqrt{\Delta x^{2}+\Delta y^{2}+\Delta z^{2}}$, the horizontal range $R_{xy}=\sqrt{\Delta x^{2}+\Delta y^{2}}$, and the unit line-of-sight vector $u={\left[u_{x},u_{y},u_{z}\right]}^{T}=\Delta p/R$. The nonlinear measurement model is

(4) $$z_{k}=h\left(X_{k}\right)+v_{k}=\left[\begin{array}{c} R+\Delta r\\ \arctan(\Delta y/\Delta x)+\Delta \theta \\ \arctan(\Delta z/R_{xy})+\Delta \varphi \\ (\Delta p^{T}\Delta v)/R \end{array}\right]+\left[\begin{array}{c} v_{r}\\ v_{\theta }\\ v_{\varphi }\\ v_{vr} \end{array}\right]$$

where $v_{k}∼N\left(0,R\right)$ with $R=\mathrm{diag}(\sigma_{r}^{2},\sigma_{\theta }^{2},\sigma_{\varphi }^{2},\sigma_{vr}^{2})$. All four measurement channels are jointly affected by the kinematic states and the biases; the range measurement directly shifts by Δr, the two angular measurements shift by Δθ and Δφ respectively, while the radial velocity measurement is bias-free.

The Jacobian of the measurement function with respect to the augmented state plays a central role in both the recursive filtering and the FIM-based observability analysis. It takes the form $H_{k}=\partial h/\partial X\in R^{4\times 9}$, with the following row-wise structure:

(5) $$H_{k}=\left[\begin{array}{ccccccccc} u_{x} & u_{y} & u_{z} & 0 & 0 & 0 & 1 & 0 & 0\\ -\frac{\Delta y}{R_{xy}^{2}} & \frac{\Delta x}{R_{xy}^{2}} & 0 & 0 & 0 & 0 & 0 & 1 & 0\\ -\frac{\Delta z\;\Delta x}{R^{2}R_{xy}} & -\frac{\Delta z\;\Delta y}{R^{2}R_{xy}} & \frac{R_{xy}}{R^{2}} & 0 & 0 & 0 & 0 & 0 & 1\\ \frac{\partial \dot{R}}{\partial x} & \frac{\partial \dot{R}}{\partial y} & \frac{\partial \dot{R}}{\partial z} & u_{x} & u_{y} & u_{z} & 0 & 0 & 0 \end{array}\right]$$

where the radial velocity Jacobian block for the position components is $\left[\partial \dot{R}/\partial x,\;\partial \dot{R}/\partial y,\;\partial \dot{R}/\partial z\right]=\frac{1}{R}\left(I_{3\times 3}-uu^{T}\right)\Delta v$. The structure of $H_{k}$ directly reflects the geometry of the problem: The first three columns couple position states to all four measurements; columns 4–6 couple velocity only to the radial velocity measurement; and columns 7–9 encode a one-to-one additive bias model for range, azimuth, and elevation, respectively. The fact that the angular bias columns (8 and 9) share nonzero entries only with the position columns (1–3) is the root of the identifiability difficulty analyzed in Section 3.

The estimation objective is to recover, at each time step *k*, both the target kinematic state $x_{t,k}$ and the systematic biases b from the incoming measurement stream $\left\{z_{1},\ldots ,z_{k}\right\}$. The central difficulty is that under straight-line flight the position and angular bias columns of $H_{k}$ are nearly linearly dependent: a shift in the azimuth bias can be perfectly compensated by a cross-track position adjustment, making the state-bias pair unidentifiable from measurements alone. The platform must therefore maneuver to generate the geometric diversity needed for successful bias decoupling.

## 3. Methodology

Building on the problem formulated in Section 2, this section first analyzes the state-bias coupling mechanism that limits recursive filter performance. A recursive PCRLB is then introduced as a maneuver-level diagnosis tool for evaluating theoretical estimation bounds under different platform trajectories. Finally, the core contribution—the Bias Subspace Information-Guided MAP-EKF (BI-MAP-EKF)—is presented, where a posterior window PCRLB serves as the online trigger for MAP refinement and evaluates whether the candidate window, together with the EKF prior, is reliable enough for MAP injection.

### 3.1. State Coupling Mechanism and Observability Analysis

The primary challenge of single-platform augmented bias estimation lies in the geometric coupling between the target’s physical position and the radar’s angular biases. This coupling renders the system weakly observable under certain trajectories (e.g., straight-line flight) [23].

To illustrate this coupling effect, consider the relative 2D position vector $p_{xy}={\left[\Delta x,\Delta y\right]}^{T}$ and the true azimuth angle $\theta_{true}=\arctan\left(\Delta y/\Delta x\right)$. Suppose the radar measures an azimuth $\theta_{m}=\theta_{true}+\Delta \theta_{true}$, where $\Delta \theta_{true}$ is the true bias. If the filter incorrectly estimates the azimuth bias with a small error δθ, such that $\Delta \hat{\theta }=\Delta \theta_{true}+\delta \theta$, the filter compensates by rotating the estimated position vector to match the observation $\theta_{m}$. The resulting position estimate ${\hat{p}}_{xy}$ can be expressed using a 2D rotation matrix:

(6) $${\hat{p}}_{xy}=R\left(-\delta \theta \right)p_{xy}=\left[\begin{array}{cc} \cos(\delta \theta ) & \sin(\delta \theta )\\ -\sin(\delta \theta ) & \cos(\delta \theta ) \end{array}\right]\left[\begin{array}{c} \Delta x\\ \Delta y \end{array}\right]$$

Applying the small-angle approximations cos(δθ)≈1 and sin(δθ)≈δθ, the induced position error $\delta p_{xy}$ is

(7) $$\delta p_{xy}={\hat{p}}_{xy}-p_{xy}\approx \left[\begin{array}{cc} 1 & \delta \theta \\ -\delta \theta & 1 \end{array}\right]\left[\begin{array}{c} \Delta x\\ \Delta y \end{array}\right]-\left[\begin{array}{c} \Delta x\\ \Delta y \end{array}\right]=\left[\begin{array}{c} \Delta y\\ -\Delta x \end{array}\right]\delta \theta$$

It can be seen from (7) that an azimuth bias error δθ is equivalent to a cross-track position displacement of magnitude $∥\Delta p_{xy}∥\cdot \delta \theta$ perpendicular to the line of sight. Under the idealized uniform straight-line geometry, such state bias pairs can become locally indistinguishable over a limited observation interval. The augmented system is therefore rank-deficient along this coupled direction, and no estimator can separate the true state from the coupled alternative without a change in the sensing geometry.

To quantify the theoretical lower bound under different maneuvering modes, the posterior Cramér–Rao Lower Bound (PCRLB) for discrete-time systems follows the Riccati recursion [24,25]:

(8) $$\begin{array}{cc} P_{k|k-1} & =FP_{k-1|k-1}F^{T}+Q \end{array}$$

(9) $$\begin{array}{cc} P_{k|k}^{-1} & ={\left(P_{k|k-1}\right)}^{-1}+H_{k}^{T}R^{-1}H_{k} \end{array}$$

where F=A is from (3) and $H_{k}$ is from (5). This recursion mirrors the EKF covariance propagation and provides a cumulative information bound over the full trajectory. In Section 4, it is used to compare how different platform trajectories affect the theoretical accuracy limit for bias estimation.

Critically, this bound is **global**; it aggregates past information without isolating whether the *current* window alone is informative. If recent data were collected under a weakly observable geometry, the recursive PCRLB could still appear favorable due to earlier informative measurements. The online trigger therefore requires a window-level metric, developed in the following subsection.

### 3.2. Bias Subspace Information-Guided MAP-EKF (BI-MAP-EKF)

To make the trigger consistent with the finite-window MAP refinement, we construct a posterior window PCRLB on the same candidate window used by the optimizer. This metric should not be interpreted as the pure geometric information of the window alone. It evaluates whether the selected window, together with the current EKF prior, is reliable enough to be fed back into the EKF.

*Recursive EKF Backbone*: The 9-dimensional augmented-state EKF backbone operates continuously to provide real-time tracking and propagate the statistical covariance. The complete predict and update iterations are as follows:

*Time Update (Prediction):*

(10) $$\begin{array}{cc} {\hat{X}}_{k|k-1} & =A{\hat{X}}_{k-1|k-1} \end{array}$$

(11) $$\begin{array}{cc} P_{k|k-1} & =AP_{k-1|k-1}A^{T}+Q \end{array}$$

*Measurement Update (Correction):*

(12) $$\begin{array}{cc} {\tilde{z}}_{k} & =z_{k}-h\left({\hat{X}}_{k|k-1}\right) \end{array}$$

(13) $$\begin{array}{cc} S_{k} & =H_{k}P_{k|k-1}H_{k}^{T}+R \end{array}$$

(14) $$\begin{array}{cc} K_{k} & =P_{k|k-1}H_{k}^{T}S_{k}^{-1} \end{array}$$

(15) $$\begin{array}{cc} {\hat{X}}_{k|k} & ={\hat{X}}_{k|k-1}+K_{k}{\tilde{z}}_{k} \end{array}$$

(16) $$\begin{array}{cc} P_{k|k} & =\left(I-K_{k}H_{k}\right)P_{k|k-1} \end{array}$$

where ${\tilde{z}}_{k}$ is the innovation, $S_{k}$ is the innovation covariance, and $K_{k}$ is the optimal Kalman gain.

*Complete Posterior Window PCRLB for MAP Injection*: For the candidate window $W_{k}=\left[k-N_{w}+1,k\right]$, the window-end state $X_{k}$ is selected as the MAP optimization variable. A historical measurement $z_{j}$ at time j≤k is originally a function of the historical state $X_{j}$. To express this measurement as a constraint on $X_{k}$, the deterministic part of the state transition is written in the backward form

(17) $$X_{j}≃\Phi_{j,k}X_{k}+\eta_{j,k},\;j\leq k,$$

where $\Phi_{j,k}$ maps the window-end state back to the historical time *j*, and $\eta_{j,k}$ represents the equivalent uncertainty induced by process noise over the interval [j,k]. Although $\Phi_{j,k}$ is a backward transition, the resulting Jacobian derived below is taken with respect to $X_{k}$. Therefore, the information contribution obtained in this section is still an information matrix for the window-end state.

For the constant-velocity model used in this paper, $\Phi_{j,k}$ has the same block form as the state transition matrix in (3), with the time interval replaced by $\left(t_{j}-t_{k}\right)$. The covariance of the backward equivalent error $\eta_{j,k}$ is denoted by $\Gamma_{j,k}$. In the linear time-invariant case, it can be written as

(18) $$\Gamma_{j,k}=\sum_{\ell =j}^{k-1}\Phi_{j,\ell +1}Q\Phi_{j,\ell +1}^{⊤},\;\Gamma_{k,k}=0.$$

This term accounts for the process uncertainty accumulated when an older measurement is interpreted as a constraint on the current window-end state.

Linearizing the historical measurement model around the state implied by $X_{k}$ gives

(19) $$z_{j}≃h_{j}\left(\Phi_{j,k}X_{k}\right)+H_{j}\eta_{j,k}+v_{j},$$

where

(20) $$H_{j}={\left.\frac{\partial h_{j}}{\partial X_{j}}\right|}_{{\hat{X}}_{j|k}},\;{\bar{H}}_{j,k}=\frac{\partial h_{j}}{\partial X_{k}}=H_{j}\Phi_{j,k}.$$

Here, $H_{j}$ is the local measurement Jacobian with respect to the historical state $X_{j}$, while ${\bar{H}}_{j,k}$ is the equivalent Jacobian with respect to the window-end state $X_{k}$. The effective covariance of the historical measurement constraint becomes

(21) $${\bar{R}}_{j,k}=R+H_{j}\Gamma_{j,k}H_{j}^{⊤}.$$

Therefore, the measurement information contributed by the candidate window is

(22) $$J_{\mathrm{meas},W}\left(k\right)=\sum_{j\in W_{k}}{\bar{H}}_{j,k}^{⊤}{\bar{R}}_{j,k}^{-1}{\bar{H}}_{j,k}.$$

Combining the prior information from the EKF with the window measurement information, the complete posterior window information matrix is written as

(23) $$J_{\mathrm{post},W}\left(k\right)=J_{\mathrm{prior}}\left(k\right)+J_{\mathrm{meas},W}\left(k\right),$$

where $J_{\mathrm{prior}}\left(k\right)=P_{k|k}^{-1}$ is the EKF posterior information at the window end. In practice, the EKF covariance in this problem converges to a slowly varying steady state under quasi-static geometries, so using $P_{k|k}$ rather than a strictly measurement-free prior has a negligible effect on the resulting PCRLB trends. The metric is therefore treated as a practical scheduling indicator for the tested operating regime.

The bias subspace is extracted from $J_{\mathrm{post},W}$ via the Schur complement. Partition $J_{\mathrm{post},W}$ into kinematic ($x_{t}$) and bias (b) blocks:

(24) $$J_{\mathrm{post},W}=\left[\begin{array}{cc} J_{xx} & J_{xb}\\ J_{bx} & J_{bb} \end{array}\right].$$

The posterior bias subspace information matrix is

(25) $$J_{b|x}=J_{bb}-J_{bx}J_{xx}^{-1}J_{xb},$$

and the corresponding posterior bias PCRLB is $C_{b|x}=J_{b|x}^{-1}$. The azimuth bias bound

(26) $$\sigma_{\Delta \theta }^{\mathrm{post}}=\sqrt{{\left[C_{b|x}\right]}_{2,2}}$$

is used to judge whether the candidate window provides a sufficiently reliable MAP update for injection into the EKF.

The complete MAP injection rule at time step *k* is

(27) $$\mathrm{Inject}\;\mathrm{MAP}\;\mathrm{if}\;\left\{\begin{array}{c} \mathrm{first}\;\mathrm{MAP}:\;T_{W}\left(k\right)\geq T_{\mathrm{init}},\\ \mathrm{subsequent}\;\mathrm{MAP}:\;\left(\sigma_{\Delta \theta }^{\mathrm{post}}\leq \tau_{\theta }\;\vee \;T_{W}\left(k\right)\geq W_{\max}\right)\;\wedge \;\left(t_{k}-t_{\mathrm{last}}\geq \Delta t_{\min}\right), \end{array}\right.$$

where $T_{W}\left(k\right)$ is the duration of the current candidate MAP window, $W_{\max}$ is the maximum allowed window time, $T_{\mathrm{init}}$ is the minimum initial window time, and $\Delta t_{\min}$ is the cooldown interval.

The three terms in (27) serve distinct roles:

- *PCRLB threshold $\tau_{\theta }$* evaluates whether the candidate window, together with the EKF prior, provides sufficient azimuth-bias information. When $\sigma_{\Delta \theta }^{\mathrm{post}}$ falls below $\tau_{\theta }$, the candidate window is considered sufficiently informative for MAP injection.
- *Maximum-window trigger $W_{\max}$* prevents indefinite postponement when the geometry remains persistently weak. If the candidate window reaches $W_{\max}$ before the PCRLB threshold is satisfied, MAP is activated by the maximum-window safeguard.
- *Cooldown interval $\Delta t_{\min}$* limits MAP frequency when the PCRLB condition is repeatedly satisfied under highly informative maneuvers.

Based on empirical tuning (Section 4), the parameters are set to $\tau_{\theta }=0.06{}^{\circ}$, $\Delta t_{\min}=15$ s, $W_{\max}=80$ s (including 20 s overlap), and the first MAP is forced at $T_{\mathrm{init}}=30$ s with checks performed every 5 s.

**MAP Optimization with EKF Prior**: When the trigger conditions are satisfied, the MAP estimation maximizes the posterior probability of the window-end state $X_{k}$ given the windowed measurements $Z_{k-N_{w}:k}$,

(28) $${\hat{X}}_{MAP}=\arg\max_{X_{k}}p\left(X_{k}|Z_{k-N_{w}:k}\right)\propto \arg\max_{X_{k}}\left(p\left(Z_{k-N_{w}:k}|X_{k}\right)\;p\left(X_{k}\right)\right),$$

where ${\hat{X}}_{k|k}$ and $P_{k|k}$ from the EKF serve as the Gaussian prior. Converting to a nonlinear least-squares cost yields

(29) $${\hat{X}}_{MAP}=\arg\min_{X_{k}}\left\{\frac{1}{2}∥X_{k}-{\hat{X}}_{k|k}{∥}_{P_{k|k}^{-1}}^{2}+\frac{1}{2}\sum_{i=k-N_{w}}^{k}{∥z_{i}-h_{i}\left(X_{k}\right)∥}_{R^{-1}}^{2}\right\}.$$

The cost is minimized via Gauss–Newton iteration. Upon convergence, the posterior covariance is

(30) $$P_{MAP}={\left(H_{aug}^{T}R_{aug}^{-1}H_{aug}+P_{k|k}^{-1}\right)}^{-1},$$

where $H_{aug}$ stacks the window Jacobians and $R_{aug}$ is the block-diagonal measurement noise covariance. Including $P_{k|k}^{-1}$ is essential. Using only the measurement FIM may underestimate the uncertainty of bias components weakly constrained within the window, which can lead to over-confident covariance injection into the subsequent EKF recursion.

**Figure 1:**
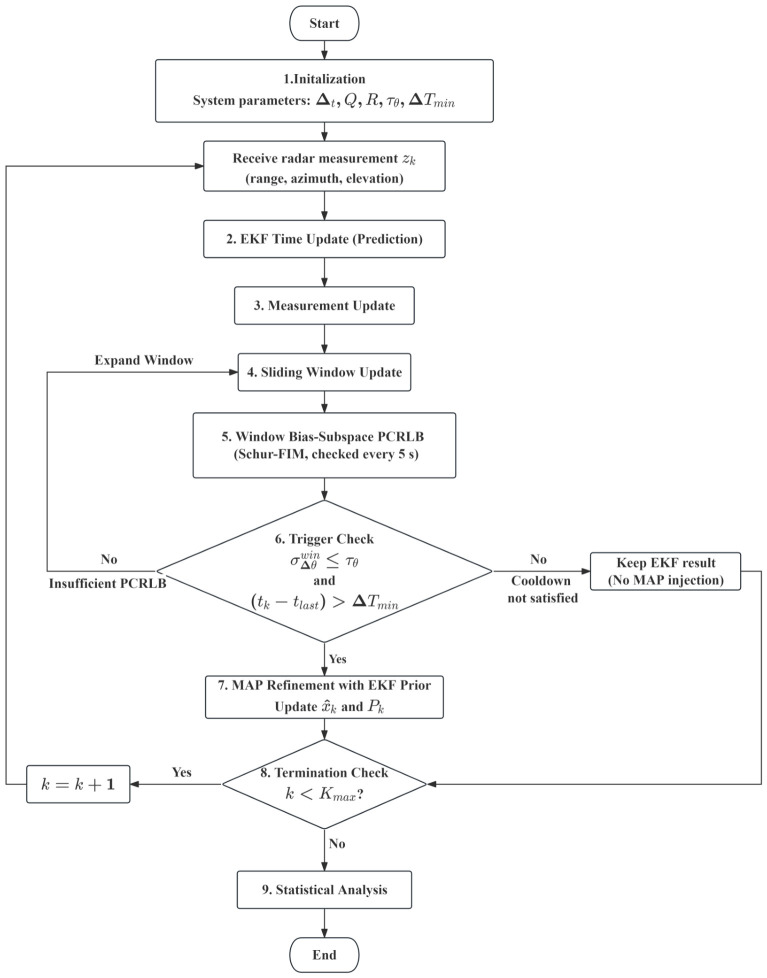
Workflow of the Bias Subspace Information-Guided maximum a posteriori (MAP)–extended Kalman filter (EKF), abbreviated BI-MAP-EKF. The EKF runs continuously while the posterior Cramér–Rao lower bound (PCRLB) is checked at admissible instants. MAP refinement is performed under the initialization, PCRLB, or maximum-window condition, subject to cooldown, and the optimized state and covariance are then injected into the EKF.

The complete pipeline of the proposed BI-MAP-EKF is summarized in Figure 1. The EKF runs at every filtering step, while the posterior window PCRLB is evaluated only at admissible check instants. When either the PCRLB condition or the maximum-window condition is satisfied after the cooldown interval, MAP refinement is performed, and the optimized state-covariance pair is injected back into the EKF.

In the implementation, ${\bar{H}}_{j,k}$ is evaluated directly by using the relative time offset $t_{j}-t_{k}$, which is equivalent to applying the chain rule $H_{j}\Phi_{j,k}$.

### 3.3. Computational Complexity

Let *n* be the dimension of the augmented state, *m* be the number of measurements per time step, $N_{w}$ be the number of measurements in the MAP window, and *I* be the number of Gauss–Newton iterations. The EKF recursion has a per-step cost dominated by the covariance propagation and update. Since n=9 and m=4 in the considered problem, these operations are nearly constant in practice.

Let $N_{c}$ denote the number of admissible PCRLB-check instants, and let $N_{M}$ denote the number of accepted MAP refinements. Beyond the EKF recursion, BI-MAP-EKF has two additional costs. First, at each check instant, the posterior window PCRLB must be evaluated by accumulating the equivalent Jacobians and effective covariance terms over the candidate window. This information-evaluation layer has a cost of approximately $O(N_{w}mn^{2})$ per check and is incurred even when MAP is not finally accepted. Second, once a MAP update is accepted, the Gauss–Newton solver requires residual and Jacobian accumulation over the selected window and solution of the n×n normal equation, with cost approximately $O(IN_{w}mn^{2}+In^{3})$.

Thus, the additional cost can be summarized as

(31) $$O\;\left(N_{c}N_{w}mn^{2}\right)+O\;\left(N_{M}\left(IN_{w}mn^{2}+In^{3}\right)\right).$$

The first term corresponds to window information evaluation, and the second term corresponds to accepted MAP optimization. Therefore, under the same number of MAP refinements, BI-MAP-EKF may still have extra PCRLB-checking overhead. Its runtime benefit mainly comes from reducing unnecessary MAP optimizations through scheduling, rather than from a cheaper MAP solver.

## 4. Simulation Part I: Observability Evaluation and Method Validation

Simulation experiments are conducted in two stages. First, maneuver-dependent observability characteristics are evaluated using a recursive PCRLB analysis, which provides a theoretical diagnosis of how different platform trajectories influence the estimability of radar biases. Second, the proposed Bias Subspace Information-Guided MAP-EKF (BI-MAP-EKF) is examined through a representative single-run experiment and a Monte Carlo comparison of different MAP scheduling strategies. The former illustrates the correction mechanism in detail, whereas the latter evaluates how information-guided scheduling changes estimation accuracy, MAP call frequency, and runtime relative to fixed-period alternatives. The core simulation parameters are summarized in Table 1. These settings are reported explicitly to make the radar sensing noise model, MAP-window configuration, and online fusion procedure reproducible. All simulations are performed in MATLAB R2022a on an Intel Core i7-13790F processor.

### 4.1. Observability Comparison Under Different Maneuvering Modes

**Figure 2:**
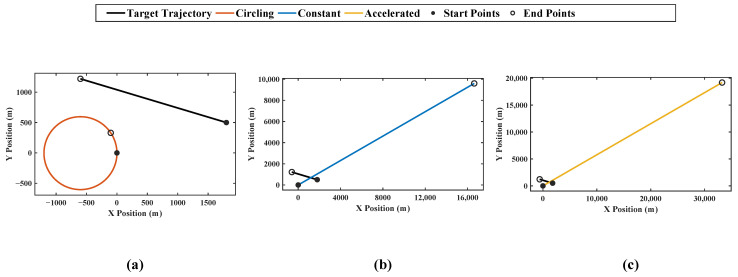
Platform and target trajectories used for maneuver-level PCRLB analysis: (**a**) circling; (**b**) constant-velocity straight-line motion; and (**c**) accelerated straight-line motion. Filled and open markers denote the start and end positions, respectively.

To clarify how platform maneuvering affects bias-state observability, three representative trajectories are compared: circling, constant-velocity straight-line, and accelerated straight-line, as shown in Figure 2. These trajectories provide different levels of geometric diversity and are used as typical diagnostic cases rather than an exhaustive set of possible flight profiles.

**Figure 3:**
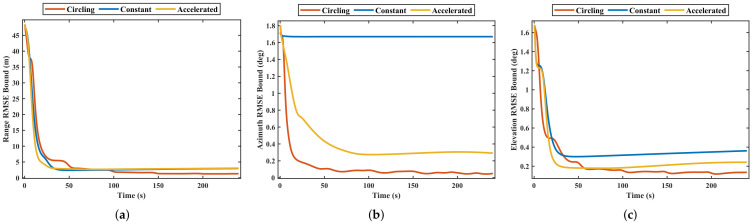
Recursive PCRLB evolution of (**a**) range bias, (**b**) azimuth bias, and (**c**) elevation bias under the three platform maneuvers. Lower bounds indicate stronger trajectory-induced identifiability.

The recursive PCRLB results in Figure 3 show that the range bias is reduced rapidly in all three modes, which is consistent with its direct additive coupling to the range measurement. The azimuth bias, however, is much more sensitive to the platform trajectory. Under constant-velocity flight, the azimuth bound remains high because the azimuth bias and the cross-track target position are difficult to decouple. Accelerated straight-line motion provides partial improvement by changing the relative geometry over time, whereas circling leads to the fastest decrease due to continuous line-of-sight rotation. The elevation bias shows an intermediate trend. These results indicate that the azimuth bias is the most geometry-sensitive component, which motivates its use as the primary indicator in the MAP-window scheduling rule.

This recursive PCRLB is used only as a maneuver-level diagnostic tool. It explains why geometric excitation is important for bias identifiability, but it is not directly used as the online MAP trigger. The trigger itself is based on the posterior window PCRLB introduced in Section 3.2.

### 4.2. Single-Run Demonstration Under Two Maneuver Scenarios

To illustrate how the proposed method behaves under qualitatively different observability conditions, two representative single-run experiments are presented: Scenario A employs a mild heading-change maneuver, while Scenario B follows an aggressive accelerating straight-line profile with stronger observability. The proposed method uses the balanced parameter set determined in Section 4.3: $\tau_{\theta }=0.06{}^{\circ}$, $\Delta t_{\min}=15$ s, and $W_{\max}=80$ s (including 20 s overlap). The scheduling metric is the complete posterior window PCRLB described in Section 3.2, which combines the EKF prior with process noise-consistent window measurement information and extracts the azimuth bias bound through the Schur complement. In the scheduling figures, three trigger types are distinguished by color and marker: initialization trigger (blue diamond), PCRLB trigger (teal circle), and maximum-window trigger (amber square).

In the scheduling subfigures, the upper panel shows the posterior azimuth bias PCRLB evaluated at the admissible check instants. The dashed horizontal line denotes the threshold $\tau_{\theta }$. The lower panel shows the MAP windows actually used for refinement; each horizontal bar represents the time span of one accepted MAP window, and the marker at the right end indicates the injection time and trigger type. A PCRLB trigger means that $\sigma_{\Delta \theta }^{\mathrm{post}}\leq \tau_{\theta }$, whereas a maximum-window trigger means that the candidate window has reached the maximum allowed time $W_{\max}$ before the PCRLB condition is satisfied.

#### 4.2.1. Scenario A: Mild-Maneuver Experiment

Scenario A is used as a weak and uneven information case. The platform performs only a mild heading change, so the angular bias information is accumulated gradually and unevenly. This scenario examines the conservative side of the scheduler; when the angular information grows slowly, the method should avoid early low-information injection, but it should also avoid waiting indefinitely.

**Figure 4:**
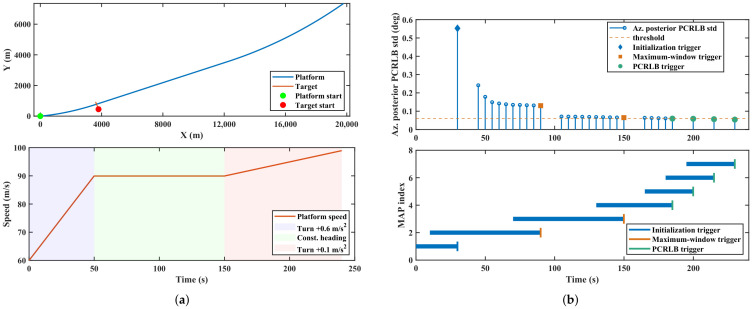
Scenario A (mild maneuver): (**a**) platform target geometry and speed profile; (**b**) posterior azimuth bias PCRLB at the admissible check instants (upper) and accepted MAP windows (lower). Horizontal bars show the window spans, and right-end markers identify the injection times and trigger types.

As shown in Figure 4, the posterior azimuth bias bound decreases only gradually after the initial maneuver. The first MAP update is forced after the minimum initial window. Among the six subsequent updates, four refinements are triggered by the PCRLB condition, while two updates are activated by the maximum-window trigger. This behavior indicates that the maximum-window safeguard remains necessary when the posterior bound does not fall below the threshold within the allowed window length.

**Figure 5:**
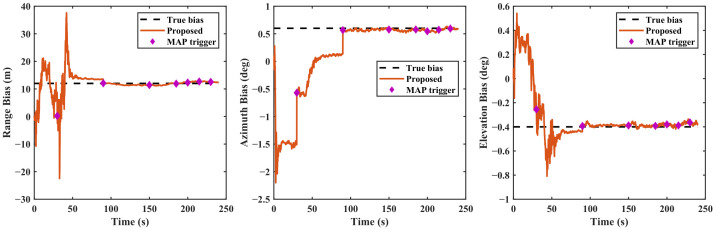
Single-run estimates of the range, azimuth, and elevation biases in Scenario A. Magenta markers denote accepted MAP injection instants.

Figure 5 shows that the range and elevation biases settle relatively quickly, whereas the azimuth bias requires a longer transient. This is consistent with the weaker angular observability in Scenario A and explains why the azimuth bias PCRLB is used as the scheduling indicator.

#### 4.2.2. Scenario B: Strong-Maneuver Experiment

Scenario B is used as a more informative case. The stronger acceleration profile alters the relative geometry more clearly, so the posterior azimuth bias bound is expected to satisfy the PCRLB threshold more regularly. This case highlights the role of the cooldown interval when the information condition is frequently met.

**Figure 6:**
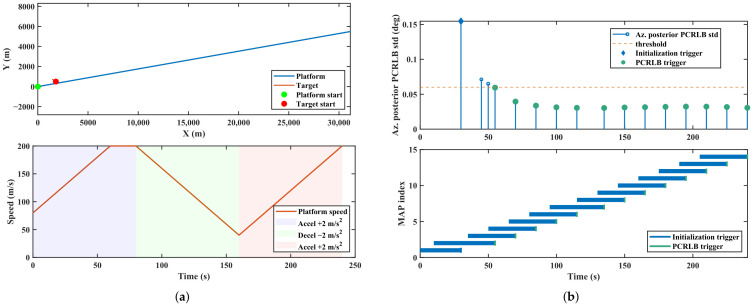
Scenario B (strong maneuver): (**a**) platform target geometry and speed profile; (**b**) posterior azimuth bias PCRLB at the admissible check instants (upper) and accepted MAP windows (lower). Horizontal bars show the window spans, and right-end markers identify the injection times and trigger types.

Figure 6 shows that the posterior azimuth bias bound falls below the threshold at nearly every admissible check. Therefore, no maximum-window trigger activation is needed in this scenario. The accepted MAP updates are mainly limited by the cooldown interval, which prevents excessive optimization when the information condition is repeatedly satisfied.

**Figure 7:**
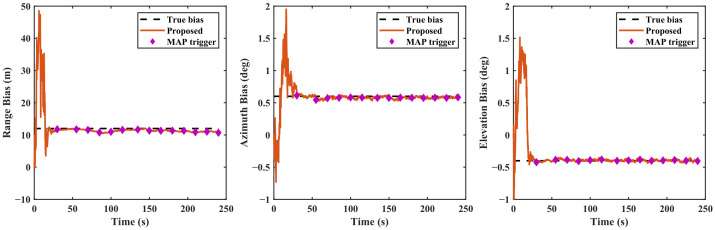
Single-run estimates of the range, azimuth, and elevation biases in Scenario B. Magenta markers denote accepted MAP injection instants.

The bias estimates in Figure 7 converge faster than in Scenario A because the maneuver provides stronger geometric excitation. Together, the two scenarios show the two roles of the proposed scheduler: the maximum-window trigger prevents indefinite waiting under weak information, while the cooldown interval limits unnecessary MAP calls under informative motion.

Table 2 summarizes the single-run bias accuracy and trigger composition of the two demonstration scenarios. The bias RMSEs are computed over the final 50 s, so they reflect the post-convergence behavior rather than the initial transient. Scenario A requires maximum-window activations, indicating that the mild maneuver does not always satisfy the PCRLB threshold before the window reaches $W_{\max}$. In contrast, Scenario B is dominated by PCRLB-triggered updates, and the shorter average window length shows that informative measurements arrive more regularly. The difference between the two rows explains why the same scheduler behaves differently in the two cases: in Scenario A it sometimes waits until the window reaches $W_{\max}$, whereas in Scenario B the PCRLB condition is usually satisfied before the maximum-window limit is reached.

### 4.3. Parameter Analysis and Strategy Comparison

#### 4.3.1. Parameter Sensitivity Analysis

The scheduler parameters are examined through 50-run Monte Carlo sweeps. In all sweeps, the same complete posterior window PCRLB as in Section 3.2 is used. The sensitivity analysis provides a trend analysis for each parameter under the tested ranges rather than a global optimum search. In each sweep, one parameter is varied while the other two are held at their baseline values ($\Delta t_{\min}=15$ s, $W_{\max}=80$ s, $\tau_{\theta }=0.06{}^{\circ}$). The tested ranges are chosen according to online implementation constraints and the physical meaning of each parameter. In particular, an overly large maximum window increases latency, computational load, and the risk of model mismatch within the constant-velocity target model; an overly small cooldown interval may cause excessive MAP refinements under informative maneuvers; and an overly loose PCRLB threshold may accept short windows with weaker bias state separation. Therefore, when the best value within a scanned range appears at a boundary, it should be interpreted as a trend under the tested constraints rather than as evidence of a global optimum.

**Figure 8:**
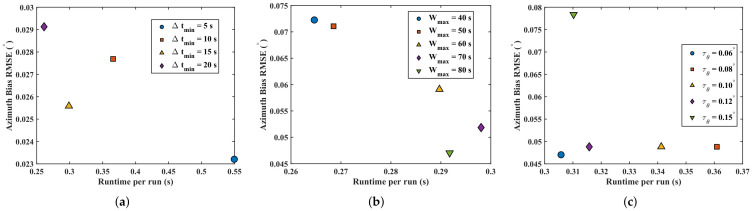
Accuracy–runtime trade-off across the three parameter sweeps: (**a**) cooldown $\Delta t_{\min}$ sweep; (**b**) max window $W_{\max}$ sweep; (**c**) threshold $\tau_{\theta }$ sweep. Each marker corresponds to one tested parameter value.

The accuracy–runtime trade-off for each sweep is shown in Figure 8. In the maximum-window sweep (Scenario A), $W_{\max}=80$ s achieves the best azimuth accuracy within the tested delay limit, suggesting that the weak-information case benefits from longer information accumulation within the tested range. In the cooldown sweep (Scenario B), $\Delta t_{\min}=5$ s gives the lowest azimuth RMSE but at 38.0 MAP calls per run, whereas $\Delta t_{\min}=15$ s reduces this to 14.0 calls with only a 0.0024° accuracy loss, showing how the update frequency can be limited under strong maneuvering. The selected setting is therefore treated as a practical operating point under the online latency and model validity constraints, rather than as a global optimum. The numerical results of all tested parameter values are summarized in Table 3.

#### 4.3.2. Comparison with Periodic MAP Baselines

Based on the sensitivity analysis, a balanced parameter set is selected: $\Delta t_{\min}=15$ s, $W_{\max}=80$ s, and $\tau_{\theta }=0.06{}^{\circ}$. This configuration is evaluated against three periodic MAP baselines (10 s/30 s, 20 s/30 s, and 10 s/50 s) and EKF only over 50 MC runs on the mild maneuver trajectory.

In general, increasing the MAP update frequency or enlarging the optimization window tends to improve estimation accuracy because the former applies corrections more frequently and the latter accumulates more measurement information. Both changes, however, increase computational cost or update latency. The three periodic baselines are therefore arranged as controlled comparisons. Per 10 s/30 s and Per 20 s/30 s use the same 30 s window and isolate the effect of update frequency, whereas Per 10 s/30 s and Per 10 s/50 s use the same 10 s interval and isolate the effect of window length. Per 10 s/50 s represents a relatively aggressive high-frequency, long-window configuration. This design allows us to examine whether the gain of BI-MAP-EKF comes simply from more frequent or longer optimization or from selecting the MAP injection times according to the posterior window PCRLB.

**Figure 9:**
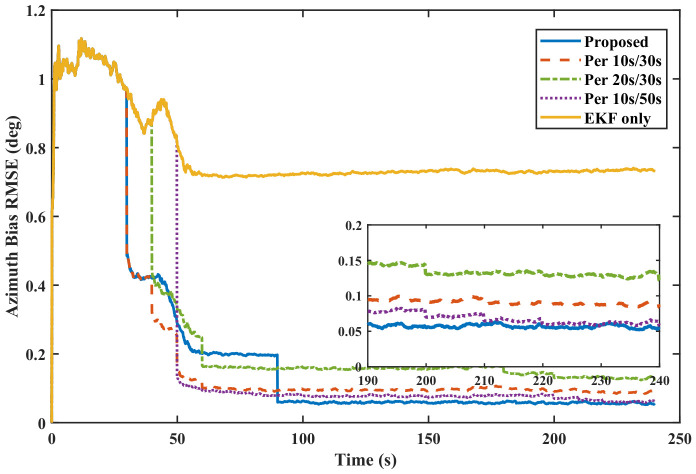
Ensemble azimuth-bias RMSE over 50 Monte Carlo runs for BI-MAP-EKF, three periodic MAP configurations, and EKF-only in Scenario A. The inset enlarges the final 50 s.

Table 4 reports the quantitative results, and Figure 9 shows the corresponding azimuth-RMSE evolution. The periodic baselines follow the expected trend. With the window fixed at 30 s, shortening the update interval from 20 s to 10 s reduces the azimuth RMSE from 0.105° to 0.076° but increases the MAP calls from 11 to 22 and the runtime from 0.17 s to 0.31 s. With the interval fixed at 10 s, enlarging the window from 30 s to 50 s further reduces the azimuth RMSE to 0.056°, while increasing the runtime to 0.45 s. These results confirm that higher update frequency and longer windows can improve accuracy, but at additional computational cost.

BI-MAP-EKF achieves an azimuth RMSE of 0.047° with 7 accepted MAP updates. Compared with the aggressive Per 10 s/50 s baseline, it provides lower azimuth error with fewer MAP calls and lower runtime. Compared with Per 10 s/30 s, it achieves better accuracy and fewer MAP calls at a similar runtime. In contrast, Per 20 s/30 s remains faster because it uses short, infrequent optimization, but its azimuth error is substantially larger. Therefore, the proposed method should not be interpreted as universally faster than periodic MAP. Its time advantage appears mainly relative to high-frequency and/or long-window optimization, while its broader benefit is the selective use of MAP when the available window is informative. The RMSE evolution in Figure 9 also shows that the largest difference between the scheduling strategies appears in the azimuth channel, which is the dominant coupled direction in the mild-maneuver case.

## 5. Simulation Part II: MHE and MAP Strategy Comparison

This section compares BI-MAP-EKF with three optimization-based baselines under the two maneuver scenarios introduced in Section 4.2. The two MHE variants represent fixed-lag refinement with different update frequencies and window lengths: MHE 5 s/50 s is an aggressive setting with frequent long-window optimization, whereas MHE 15 s/30 s is a lighter setting with fewer updates and a shorter window. The periodic MAP baseline uses the same augmented state model but injects MAP refinements at a fixed interval, without checking whether the accepted window is informative for azimuth bias separation. A full-batch factor graph smoother is not included as a direct online baseline because it solves a different smoothing problem and may use measurements beyond the current filtering instant. The two fixed-lag MHE configurations are therefore used as causal smoothing baselines under the same measurement history. Incremental factor graph implementations will be considered in future work.

All methods use the same radar measurement sequences and the same target bias state definition. The comparison is therefore not intended to show that one optimizer is universally better in every channel. Instead, it examines whether the proposed scheduler can use fewer accepted MAP refinements while preserving the accuracy gains that matter most for this problem, namely azimuth bias and horizontal position estimation.

### 5.1. Scenario A: Mild-Maneuver Comparison

Scenario A uses the mild heading-change trajectory. This is the more difficult case for online bias calibration because the angular information is not distributed uniformly over time. The range bias is directly observed through the range channel, but the azimuth bias is mainly separated from horizontal target position when the platform geometry changes. As a result, a fixed-period optimizer may refine the estimate even when the current window adds little new azimuth bias information.

This scenario is therefore used as the main test for the proposed scheduling idea. If the PCRLB-based window check is meaningful, the method should not simply increase the number of MAP refinements. It should instead reject or postpone low-information windows and keep the useful correction effect of long-window optimization with fewer accepted MAP calls.

**Figure 10:**
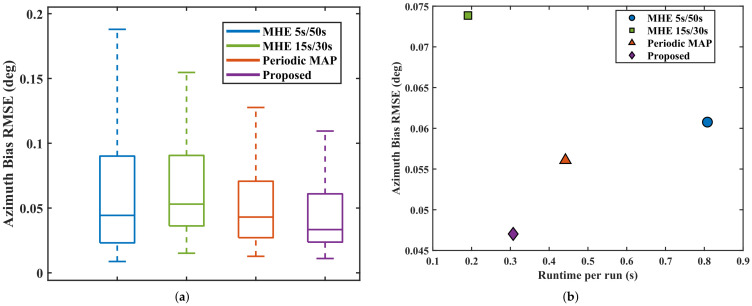
Scenario A (mild maneuver): (**a**) distribution of the per-run azimuth-bias RMSE; (**b**) mean azimuth bias RMSE versus runtime for each method.

Figure 10 gives a compact view of the azimuth error distribution and the runtime–accuracy trade-off, while Table 5 lists the full bias and position metrics. The main gain appears in the azimuth and horizontal-position channels. This is consistent with the coupling analysis in Section 3.1: The dominant ambiguity in this trajectory is not the range bias, but the trade-off between azimuth bias and cross-track target position.

The two MHE settings illustrate the accuracy–cost tension. MHE 5 s/50 s performs frequent long-window refinements and therefore keeps a good azimuth estimate, but the number of optimizations is high. MHE 15 s/30 s reduces runtime, but the shorter and less frequent windows provide weaker bias state separation in this trajectory. Periodic MAP 10 s/50 s benefits from a long window, but it still refines according to elapsed time rather than window informativeness. BI-MAP-EKF differs from these baselines by accepting fewer but more selective MAP updates. Its advantage in Scenario A should therefore be interpreted as a scheduling gain under weak and uneven observability rather than as a cheaper MAP solver. Its runtime is lower than that of the aggressive MHE 5 s/50 s and Periodic MAP 10 s/50 s baselines but higher than that of the lightweight MHE 15 s/30 s configuration. Therefore, no universal runtime advantage is claimed; the gain lies in obtaining better azimuth/XY accuracy with fewer accepted MAP refinements under weak and uneven information.

### 5.2. Scenario B: Strong-Maneuver Comparison

Scenario B uses the accelerating straight-line platform. Compared with Scenario A, the stronger maneuver produces informative windows more regularly. This changes the role of the scheduler. In Scenario A, the main risk is accepting weak windows too early. In Scenario B, the main risk is the opposite: The PCRLB condition can be satisfied frequently, so the cooldown interval becomes important for limiting unnecessary refinements.

For this reason, Scenario B is not expected to produce the same relative advantage as Scenario A. When useful information is available in most windows, a fixed-period MAP strategy is less likely to choose a severely poor window, and aggressive MHE can also benefit from frequent refinement. This scenario is included to check whether BI-MAP-EKF remains competitive when the observability condition is already favorable.

**Figure 11:**
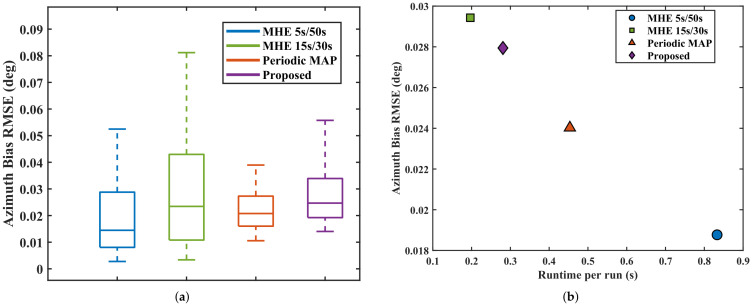
Scenario B (strong maneuver): (**a**) distribution of the per-run azimuth bias RMSE; (**b**) mean azimuth bias RMSE versus runtime for each method.

The results in Figure 11 and Table 6 follow this expectation. MHE 5 s/50 s obtains the smallest azimuth RMSE, which is reasonable because it applies the most aggressive long-window refinement. The periodic MAP baseline is also strong in this scenario, since fixed intervals are less harmful when informative windows occur regularly. BI-MAP-EKF does not achieve the minimum azimuth RMSE here. Its role is better described as a lower-call alternative; it keeps the azimuth error in a competitive range, gives the lowest XY position error among the tested methods, and uses fewer accepted MAP refinements than the high-rate MHE and periodic MAP settings. Its runtime is lower than those of MHE 5 s/50 s and Periodic MAP 10 s/50 s but not lower than that of the lightweight MHE 15 s/30 s setting.

This result also clarifies the boundary of the proposed scheduler. When computation is unconstrained and the geometry is consistently informative, aggressive smoothing can obtain a slightly better azimuth estimate. The proposed method is designed for online operation, where every MAP injection has to be justified by its information contribution and computational cost. Therefore, the value of BI-MAP-EKF in Scenario B lies less in absolute azimuth dominance and more in maintaining competitive azimuth/XY accuracy with fewer refinements than the high-frequency, long-window baselines.

Taken together, the two scenarios show that the clearest benefit of BI-MAP-EKF appears when the information content of the candidate window varies over time. In the mild-maneuver case, the posterior PCRLB check postpones or rejects low-value refinements and retains the accuracy benefit of long-window MAP with fewer accepted updates. In the strong-maneuver case, informative windows occur regularly, so fixed-period MAP and aggressive MHE are already competitive; the cooldown interval then mainly prevents the scheduler from becoming an unnecessarily frequent optimizer.

BI-MAP-EKF should therefore be interpreted as an information-aware MAP scheduler rather than a universally more accurate or faster optimizer. Its runtime benefit appears only when the reduction in MAP optimizations is large enough to offset the additional posterior-PCRLB evaluation, particularly relative to high-frequency and/or long-window baselines. Its primary purpose is to determine when MAP refinement is worth injecting into a recursive online estimator.

## 6. Discussion

The simulation results support the proposed scheduling idea, but the current validation is mechanism-level rather than field-level. Public airborne radar datasets rarely provide synchronized range, azimuth, elevation, and Doppler measurements together with independently calibrated systematic-bias ground truth, which limits direct evaluation of online bias registration. Future work will therefore include higher-fidelity measurement models, hardware-in-the-loop tests, and real-data validation when suitable calibration references are available. One technical limitation is that the posterior window PCRLB computation uses the current EKF covariance as a prior. When the EKF has not yet converged, the prior may be overly diffuse, making the PCRLB metric too conservative. The initialization trigger at $T_{\mathrm{init}}=30$ s partially compensates for this, but a more adaptive prior-scaling strategy could be considered. Under non-Gaussian noise or intermittent outliers, the quadratic MAP cost may produce over-weighted residuals; robust loss functions or measurement quality gating would be required.

The present PCRLB trigger is derived under a CV target model, Gaussian measurement noise, a slowly varying bias model, and accurate platform state assumptions. If the target performs abrupt maneuvers, the MAP window may contain model mismatch and the PCRLB may become overconfident. If radar biases change abruptly, the random-walk model may delay adaptation, and a change detector or IMM-type bias model would be needed. If the platform INS/GNSS solution contains non-negligible errors, the platform state uncertainty should be augmented into the state or folded into the measurement covariance.

Another limitation is that the parameter tuning relies on empirical sweeps. The scheduling parameters are not claimed to be globally optimal; they are selected from bounded ranges that reflect the intended online operating regime. Extending these ranges may further improve one metric, such as steady-state RMSE or runtime, but it would also change the latency, responsiveness, or model validity assumptions of the estimator. The sensitivity analysis in Section 4.3 clarifies the main accuracy, runtime, and update frequency trade-offs around the selected values, but the preferred operating point may change for substantially different platform dynamics or sensor characteristics. A systematic, context-aware parameter selection guideline would improve practical applicability.

Finally, the current evaluation is limited to simulated trajectories with a single non-maneuvering target. For other flight geometries, the expected scheduling behavior follows the same principle: Slowly varying line-of-sight geometry tends to produce longer accepted windows or maximum-window activations, whereas persistently informative geometry shifts the main scheduling role to the cooldown interval. In multi-target or cluttered environments, data association errors could corrupt the MAP window and degrade the trigger decisions. Extending the framework to incorporate measurement-to-target assignment uncertainty is a direction for future work.

## 7. Conclusions

The results suggest that the posterior window PCRLB is useful for deciding when MAP refinement should be injected into a recursive EKF. The benefit is most visible when the available geometry is weak or uneven, where fixed-period optimization may spend updates on windows with limited azimuth bias information. Under stronger maneuvering, aggressive MHE can still obtain the smallest azimuth RMSE, but the proposed scheduler provides a lower-call alternative with competitive accuracy. Given that posterior window PCRLB evaluation introduces additional computation, a runtime advantage is expected only when the resulting reduction in MAP refinements offsets this overhead, as observed relative to the high-frequency or long-window baselines. Future work will focus on real-data validation, abrupt bias changes, and extensions to platform-state uncertainty.

## Figures and Tables

**Table 1:** Radar/process-noise and Bias Subspace Information-Guided maximum a posteriori–extended Kalman filter (BI-MAP-EKF) scheduling parameters.

Parameter	Value
Range Noise ($\sigma_{r}$)	5.0 m
Azimuth Noise ($\sigma_{\theta }$)	0.12 deg
Elevation Noise ($\sigma_{\varphi }$)	0.12 deg
Radial Velocity Noise ($\sigma_{vr}$)	0.6 m/s
Sampling Interval (Δt)	0.1 s
Maximum MAP window time ($W_{\max}$)	80 s (including 20 s overlap)
MAP Cooldown Interval ($\Delta t_{\min}$)	15 s
MAP Trigger Threshold ($\tau_{\theta }$)	0.06°
Initialization MAP trigger ($T_{\mathrm{init}}$)	30 s
PCRLB check interval	5 s
Maximum MAP Iterations	8
**Process noise (discrete-time, Δt=0.1 s)**	
Target accel. std ($\sigma_{a}$)	0.5 m/s^2^ (Scenario A)/0.4 m/s^2^ (Scenario B)
Range bias RW std ($\sigma_{b_{r}}$)	0.05 m
Azimuth bias RW std ($\sigma_{b_{\theta }}$)	0.005°
Elevation bias RW std ($\sigma_{b_{\varphi }}$)	0.005°

**Table 2:** Final-50-s single-run bias root-mean-square error (RMSE) and MAP-trigger statistics for Scenarios A and B.

Scenario	Range RMSE (m)	Azimuth RMSE (deg)	Elevation RMSE (deg)	Init. Trigger	PCRLB Trigger	Max-Win. Trigger	Total MAP	Avg. Win. Len. (s)
Scenario A	0.56	0.036	0.027	1	4	2	7	49.9
Scenario B	0.98	0.023	0.015	1	13	0	14	35.6

**Table 3:** Sensitivity of estimation accuracy, MAP calls, and runtime to the three scheduling parameters over 50 Monte Carlo runs. Boldface marks the best value within each sweep, and the selected operating point is listed in the last column.

Sweep	Scn.	Value	Az. RMSE (deg)	XY RMSE (m)	MAP /Run	Runtime (s)	Selected
$\Delta t_{\min}$	B	5 s	**0.023 ± 0.011**	8.64 ± 6.01	38.0	0.55	$\Delta t_{\min}=15$ s
		10 s	0.028 ± 0.011	10.06 ± 6.08	20.0	0.37	
		15 s	0.026 ± 0.009	**8.47 ± 5.27**	14.0	0.30	
		20 s	0.029 ± 0.012	10.04 ± 6.22	11.0	**0.26**	
$W_{\max}$	A	40 s	0.072 ± 0.052	17.80 ± 14.03	11.0	**0.26**	$W_{\max}=80$ s
		50 s	0.071 ± 0.048	17.32 ± 13.01	8.0	0.27	
		60 s	0.059 ± 0.045	14.44 ± 11.96	7.0	0.29	
		70 s	0.052 ± 0.033	12.17 ± 8.89	7.0	0.30	
		80 s	**0.047 ± 0.033**	**11.15 ± 8.83**	7.0	0.29	
$\tau_{\theta }$	A	0.06°	**0.047 ± 0.033**	**11.15 ± 8.83**	7.0	**0.31**	$\tau_{\theta }=0.06{}^{\circ}$
		0.08°	0.049 ± 0.033	11.72 ± 8.75	11.0	0.36	
		0.10°	0.049 ± 0.033	11.72 ± 8.75	11.0	0.34	
		0.12°	0.049 ± 0.033	11.72 ± 8.75	11.0	0.32	
		0.15°	0.078 ± 0.059	19.49 ± 15.74	13.8	0.31	

**Table 4:** Estimation RMSE and computational statistics for BI-MAP-EKF, periodic MAP configurations, and EKF-only in Scenario A over 50 Monte Carlo runs. RMSE values are reported as mean ± standard deviation.

	Method	Proposed	Per 10 s/30 s	Per 20 s/30 s	Per 10 s/50 s	EKF
Metric						
Range RMSE (m)		0.64 ± 0.42	0.76 ± 0.50	0.78 ± 0.52	0.64 ± 0.41	0.93 ± 0.68
Azimuth RMSE (deg)		0.047 ± 0.033	0.076 ± 0.050	0.105 ± 0.084	0.056 ± 0.039	0.591 ± 0.438
Elevation RMSE (deg)		0.018 ± 0.005	0.021 ± 0.008	0.028 ± 0.015	0.019 ± 0.006	0.048 ± 0.034
XY Position RMSE (m)		11.15 ± 8.83	19.30 ± 13.20	26.80 ± 22.00	13.70 ± 10.36	155.10 ± 115.40
Time/run (s)		0.31	0.31	0.17	0.45	0.03
MAP calls/run		7.0	22.0	11.0	20.0	0.0

Per 10 s/30 s: periodic MAP with 10 s interval and 30 s window; Per 20 s/30 s: 20 s interval and 30 s window; Per 10 s/50 s: 10 s interval and 50 s window.

**Table 5:** Estimation RMSE and computational statistics for moving-horizon estimation (MHE), periodic MAP, and BI-MAP-EKF in Scenario A from 50 Monte Carlo runs. RMSE values are reported as mean ± standard deviation.

	Method	MHE 5 s/50 s	MHE 15 s/30 s	Periodic MAP 10 s/50 s	Proposed
Metric					
Range RMSE (m)		0.68 ± 0.35	0.41 ± 0.32	0.64 ± 0.41	0.64 ± 0.42
Azimuth RMSE (deg)		0.061 ± 0.049	0.074 ± 0.071	0.056 ± 0.039	0.047 ± 0.033
Elevation RMSE (deg)		0.107 ± 0.065	0.020 ± 0.015	0.019 ± 0.006	0.018 ± 0.005
XY Position RMSE (m)		15.32 ± 12.54	19.22 ± 18.88	13.70 ± 10.36	11.15 ± 8.83
Runtime/run (s)		0.81	0.19	0.44	0.31
MAP calls/run		39.0	15.0	20.0	7.0

**Table 6:** Estimation RMSE and computational statistics for MHE, periodic MAP, and BI-MAP-EKF in Scenario B over 50 Monte Carlo runs. RMSE values are reported as mean ± standard deviation.

	Method	MHE 5 s/50 s	MHE 15 s/30 s	Periodic MAP 10 s/50 s	Proposed
Metric					
Range RMSE (m)		0.59 ± 0.39	0.27 ± 0.21	0.76 ± 0.50	0.77 ± 0.56
Azimuth RMSE (deg)		0.019 ± 0.013	0.029 ± 0.022	0.024 ± 0.011	0.026 ± 0.009
Elevation RMSE (deg)		0.096 ± 0.041	0.024 ± 0.015	0.017 ± 0.004	0.019 ± 0.005
XY Position RMSE (m)		8.90 ± 6.40	14.00 ± 10.00	8.70 ± 6.10	8.50 ± 5.30
Runtime/run (s)		0.82	0.19	0.45	0.29
MAP calls/run		39.0	15.0	20.0	14.0

## Data Availability

The simulation data and source code that support the findings of this study are available from the corresponding author upon reasonable request.
